# Development and validation of a pragmatic natural language processing approach to identifying falls in older adults in the emergency department

**DOI:** 10.1186/s12911-019-0843-7

**Published:** 2019-07-22

**Authors:** Brian W. Patterson, Gwen C. Jacobsohn, Manish N. Shah, Yiqiang Song, Apoorva Maru, Arjun K. Venkatesh, Monica Zhong, Katherine Taylor, Azita G. Hamedani, Eneida A. Mendonça

**Affiliations:** 1 BerbeeWalsh Department of Emergency Medicine, University of Wisconsin School of Medicine and Public Health, Madison, WI USA; 2 Health Innovation Program, University of Wisconsin–Madison, Madison, WI 53705 USA; 3 Department of Medicine, Division of Geriatrics, University of Wisconsin School of Medicine and Public Health, Madison, WI United States; 4 Department of Biostatistics and Medical Informatics, University of Wisconsin-Madison, Madison, WI USA; 5 Department of Emergency Medicine and Center for Outcomes Research and Evaluation, Yale University School of Medicine, New Haven, CT USA; 6 Department of Pediatrics and Department of Biostatistics, Indiana University School of Medicine, Indianapolis, IN USA; 7 Regenstrief Institute, Indianapolis, IN USA

**Keywords:** Falls, Electronic health record, Emergency medicine, Natural language processing, Geriatrics

## Abstract

**Background:**

Falls among older adults are both a common reason for presentation to the emergency department, and a major source of morbidity and mortality. It is critical to identify fall patients quickly and reliably during, and immediately after, emergency department encounters in order to deliver appropriate care and referrals. Unfortunately, falls are difficult to identify without manual chart review, a time intensive process infeasible for many applications including surveillance and quality reporting. Here we describe a pragmatic NLP approach to automating fall identification.

**Methods:**

In this single center retrospective review, 500 emergency department provider notes from older adult patients (age 65 and older) were randomly selected for analysis. A simple, rules-based NLP algorithm for fall identification was developed and evaluated on a development set of 1084 notes, then compared with identification by consensus of trained abstractors blinded to NLP results.

**Results:**

The NLP pipeline demonstrated a recall (sensitivity) of 95.8%, specificity of 97.4%, precision of 92.0%, and F1 score of 0.939 for identifying fall events within emergency physician visit notes, as compared to gold standard manual abstraction by human coders.

**Conclusions:**

Our pragmatic NLP algorithm was able to identify falls in ED notes with excellent precision and recall, comparable to that of more labor-intensive manual abstraction. This finding offers promise not just for improving research methods, but as a potential for identifying patients for targeted interventions, quality measure development and epidemiologic surveillance.

**Electronic supplementary material:**

The online version of this article (10.1186/s12911-019-0843-7) contains supplementary material, which is available to authorized users.

## Background

Falls among older adults are common, with one in three older adults falling each year [1]. Falls are associated with significant mortality [2], long term morbidity from injuries such as hip fractures [3, 4], and a cost of over $19 billion annually in the US alone [5]. The emergency department (ED) both cares for a large number of fall-related injuries and offers an ideal site to identify and intervene on high risk patients to prevent future falls [6]. Despite the prevalence and negative consequences of falls, identifying these events within electronic health records has been challenging [7]. A foundational step in examining falls in the ED using Electronic Health Record (EHR) data is creating a definition which captures fall patients adequately without the need for burdensome, and in many cases impractical manual chart review.

Identifying fall visits accurately in EHR data is a priority in geriatric emergency medicine research, as further research is needed to create valid and feasible approaches to both identifying adults at high risk of fall and creating interventions to mitigate that risk [8]. Furthermore, reliable identification of fall phenotype without the need for manual abstraction offers the potential to create a denominator for quality measures and surveillance to improve patient care. Previous work studying falls commonly utilizes ICD-9 and 10 diagnostic codes to identify falls in both single center and large datasets given the ready availability of diagnosis data [9–13]. Although this is a standard procedure for identifying conditions within outcomes and health services research, it may miss many patients, particularly in the ED, where fall visits may result in other diagnosis codes reflecting the injury sustained (e.g., fractures, contusions, head trauma) without mention of the mechanism of injury. Additionally, diagnosis codes could identify an underlying etiology of a fall (such as syncope) as opposed to the fall itself. This phenomenon is not unique to falls as discharge diagnoses often have poor concordance with ED patients’ reason for visit, need for admission, or further advanced care [14]. Falls offer a characteristic example of a difficult to classify “syndromic” presentation, and given their immense public health burden are an ideal use case for developing novel methods of identification.

Given the above limitations in using structured data to identify fall visits, Natural Language Processing (NLP), with the ability to more directly evaluate physician documentation, offers the promise of an improved ability to detect falls based on the narrative text included in provider notes [15]. Medical literature evaluating NLP has in many cases gravitated from simple rules-based systems to statistical methods, which offer the potential for improved generalizability and performance [16]. Unfortunately, barriers including the need for access to large curated datasets often make training these systems impractical, and have slowed widespread adoption [16]. In some contexts, rules-based NLP algorithms have performance similar to statistical approaches [17], and have been used to identify syndromes, including falls, in in large multispecialty note databases, although in this case without validation beyond calculation of a false positive rate [18, 19].

The goal of this study was to design and validate a pragmatic, rules-based NLP approach for identification of fall patients in the ED. Our rationale for choosing this approach is that 1.) falls are generally documented using only a few standard phrases, and 2.) a short rules-based algorithm would be easily adaptable between clinical sites, as well as potentially embeddable within existing EHR products.

## Methods

### Study design and setting

We performed a retrospective observational study using EHR data at a single academic medical center ED with level 1 trauma center accreditation and approximately 60,000 patient visits per year. The text of all ED provider notes recorded at visits to the University of Wisconsin Hospital ED made by patients aged 65 years or older (from 12/13/2016 through 04/24/2017) were collected in a dataset. Notes from this database were randomly selected for individual patients via algorithm without replacement (i.e. notes from within the study period were randomly selected and included in the dataset unless they were collected from a patient already represented in the database) to create separate development and test datasets, each consisting of notes from unique patients.

### Algorithm development

**Fig. 1:**
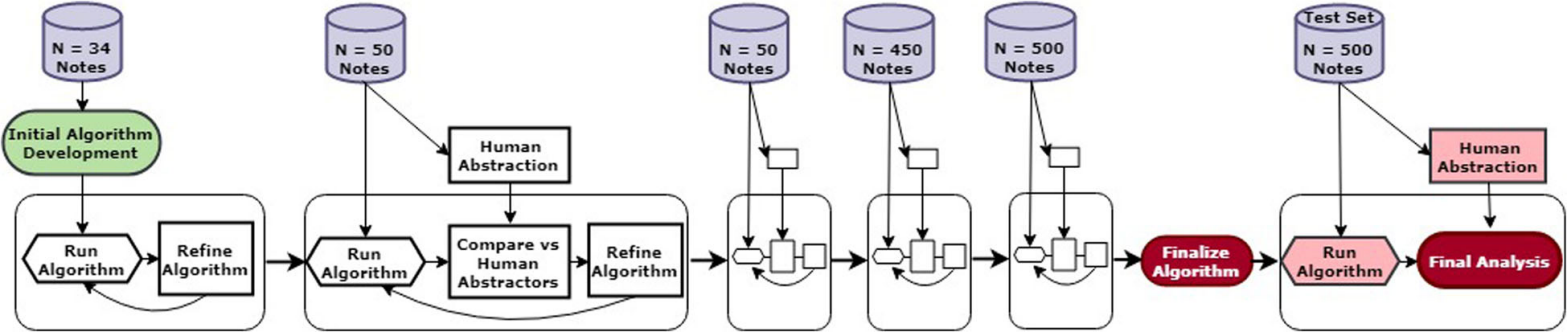
NLP Algorithm Development, Manual Abstraction, and Evaluation Process

We used Python (Python Software Foundation) to implement a pragmatic, rules-based NLP algorithm for detecting falls in ED provider notes. The algorithm was developed and refined in an iterative process; with additional notes added to the development set to refine the algorithm and improve performance until adequate performance (in this case recall and specificity both in excess of 90%) was achieved and further addition of notes seemed unlikely to generate significant increases in performance (see Fig. 1 for depiction of the algorithm development process). The algorithm was developed on a small set, with notes added in progressively larger increments. The total development set numbered 1084 at the time we believed that performance increases had plateaued and the algorithm was ready for testing. After development of the algorithm was complete, a test dataset comprised of 500 previously unused notes was randomly selected from the available visits described above. None of the notes within the testing set were part of the development set or were used to otherwise provide any input into algorithm development.

**Fig. 2:**
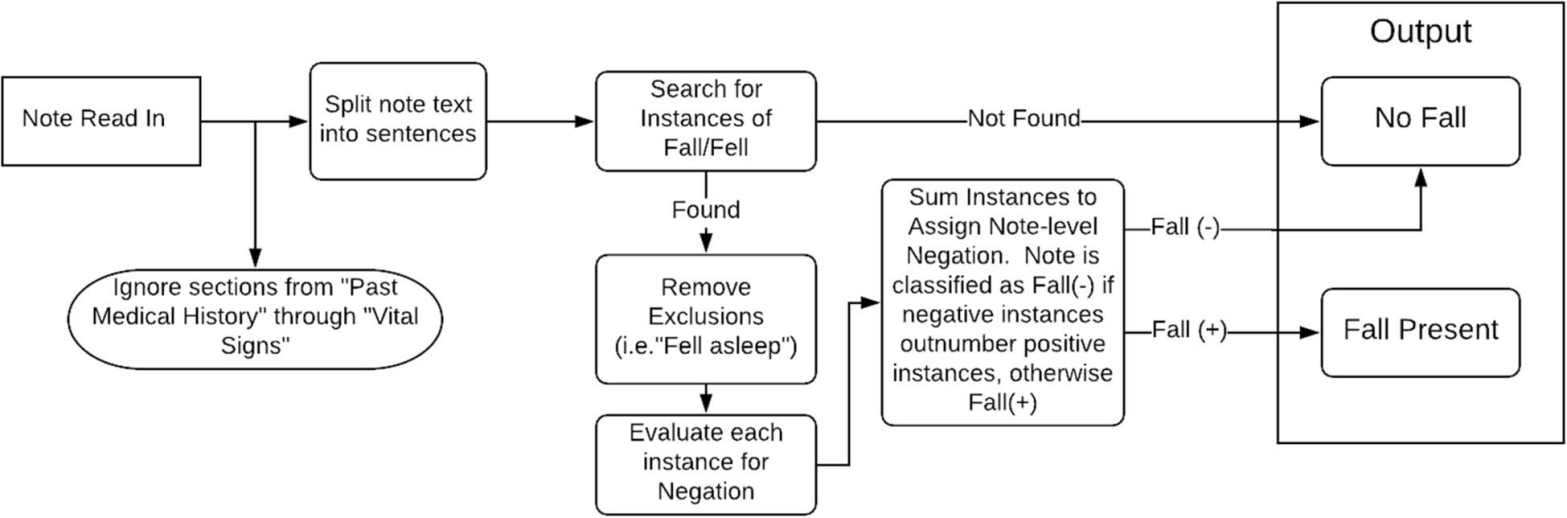
Algorithm Schematic

Figure 2 graphically describes data processing within the algorithm, and detailed algorithm notes including key python expressions are available in Additional file 1. Notes were read into the algorithm in text format, and portions of the note found during early development to be irrelevant were removed to prevent false positives. This was necessary as the “past medical history” section of notes often mentions falls preceding or unrelated to the current presentation. In addition, the “review of systems” portion of the note occasionally mentions falls amongst a long list of negated items difficult to parse based on lack of surrounding sentence structure. Once these sections were removed, the retained sections of the note were divided into sentences. Sentences were individually examined for the presence of a “fall” or “fell” term (with multiple tenses/forms using a regular expression). These fall instances were then checked against a list of exceptions, such as “fell asleep,” which were ignored. The algorithm also ignored instances when fall terms were part of a different word (e.g., “fellow”, “fallopian”). Exceptions were pre-specified in some cases, and added iteratively during development as necessary in response to specific cases in the development set. Fall mentions that involved a high degree of uncertainty (e.g., “may have fallen”, “uncertain if patient fell”), that were averted (e.g., “almost fell”), or referred to fall risks were excluded, while those using language indicating certainty for the purposes of medical decision making (e.g., “presumed fall”, “believed to have fallen”) were included.

After exceptions were removed, each sentence containing one or more fall instances were examined for negation. Negation is critical in this domain, as physicians often specifically document that a patient did not fall, or that a patient fell but had other pertinent negative findings. The program negated any instance with a negative indicator preceding the fall term in the sentence; negation terms included “no”, “not”, “n’t”, “negative”, “never”, “didn’t”, “without”, “denies”, and “deny.” Based on results during algorithm development, negation terms mentioned after a fall term were ignored. These were much more likely to refer to events that did not occur during a fall, such as “Patient fell but did not lose consciousness” or “Fell but denies striking head”.

Although the algorithm identified instances of falls in each sentence, the ultimate assessment of whether or not a fall occurred was measured at the level of the provider note (ED visit). As expected, many notes contained more than one sentence describing a fall, and/or multiple instances within a single sentence. This presented a challenge when some instances were negated and others were not. In a given note, if positive instances outnumbered negated instances, the note was coded as positive. If negated instances were equal to or outnumbered the positive instances, a note was coded as negated. When there were equal numbers of both positive and negated mentions, the note was coded as negated. This ‘tie-breaking’ decision was made based on sensitivity analysis in which we systematically examined the performance of different aggregation strategies on the development set. The tie-breaking rule that was chosen (negation-favoring) produced a more balanced distribution of false positives and false negatives, compared to positive-favoring tie-breaking strategies which generated many more false positives than false negatives (i.e., higher sensitivity at the expense of lower specificity).

### Manual abstraction

Manual abstraction was performed on all notes in the development and test sets. Data abstraction was conducted by trained nonclinical reviewers using a standardized data form (see Additional file 1). For the purpose of this study, we used the WHO definition of a fall as “an event whereby an individual unexpectedly comes to rest on the ground or another lower level” [20]. A coding manual was developed to clarify and operationalize the definition (i.e., what was and was not considered a fall). Coders were instructed that positive fall mentions had to be directly related to the reason for the current ED visit. To create a consensus code as a gold standard, all notes were coded by two reviewers, with the primary investigator and additional researcher assigning fall status by consensus in cases of disagreement. Reviewers were trained, and initial interrater reliability established, using 50 randomly selected notes from the development set during the algorithm development phase [21]. Final interrater reliability was measured on the full test set, concurrent with the running of the NLP algorithm. Abstractors for the test set were not involved in algorithm development and were unaware of NLP results as the algorithm was run on this set after consensus coding was completed.

The results generated by the NLP algorithm were compared to the gold standard consensus coding to calculate precision, specificity, recall, and F1 score of the automated method. Data were analyzed using Stata® 15 (StataCorp, College Station, TX). Data were analyzed on the basis of whether a positive fall occurrence was detected in the provider note by the algorithm and/or reviewers. While we tracked negated instances of falls, for the purposes of algorithm validation, negated fall instances (e.g., “Patient denies falls”) were categorized in the “no fall” group.

## Results

### Interrater reliability

Interrater reliability was established twice during the analysis process. The first assessment occurred at the completion of the reviewers’ manual abstraction training on a subset of 50 provider notes used during the algorithm development phase—at which point reviewers demonstrated 94.0% agreement. Reviewers also had high interrater reliability for abstraction of the full test set (*n* = 500), demonstrating 98.4% raw agreement, Kappa = .96 (std error = 0.045).

### Incidence of falls

Of the notes in the test set, 24.0% were consensus coded by reviewers (gold standard) as a positive instance of a fall (120 of 500). Reviewers also determined that 34 of the 500 notes (6.8%) contained a negated mention of a fall, indicating that no fall actually occurred even though a fall-identifying word was included. The results of the NLP algorithm indicated that 25.0% of notes in the test set were positive instances of falls (125 of 500), also with 34 notes (6.8%) containing a negated fall mention.

### NLP performance

Results comparing performance of the NLP algorithm to that of gold standard manual abstraction are presented in Tables 1 and 2. The final NLP algorithm achieved a sensitivity (recall) of 95.8% (95%CI 90.5–98.6), specificity of 97.4%, (95%CI 95.2–98.7%) a positive predictive value (precision) of 92.0% (95%CI 86.2–95.5%), a negative predictive value of 98.7% (95% CI 96.9–99.4%), in the test set. The accuracy was 97.0% (95%CI 95.1–98.3%). As depicted in Table 1, only 15 of the 500 notes (3.00%) were misclassified when compared with the gold standard human coding (with 10 false positives and 5 false negatives).

**Table 1:** Comparison of NLP Performance to Gold Standard Consensus Coding

		Gold Standard Consensus Coding		
		Fall	No Fall	Total
NLP	Fall	115	10	125
	No Fall	5	370	375
Total		120	380	500

**Table 2:** Pragmatic NLP Performance Metrics

Sensitivity (Recall)	95.8%
Specificity	97.4%
Accuracy	97.0%
PPV (Precision)	92.0%
NPV	98.7%
F1 Score	0.939

The nature of these mismatches is described in Table 3. Three of the false negative instances were the result of human reviewers detecting a fall (based on the WHO definition) when no form of the word “fall” was included in the note. One was correctly excluded by the algorithm as a past fall, but in this instance the fall directly precipitated the ED visit. The remaining false negative utilized a fall-related acronym (FOOSH for “fall on outstretched hand”) to describe the incident, without referring to a fall anywhere else in the note.

**Table 3:** Description of Errors in NLP Performance, by Error Type

Note Text^a^	Error Assessment
**False Positive**	
“Chief Complaint Patient presents with Other weakness Fall”	Chief complaint was in error. Visit was for causes unrelated to a fall (diarrhea and weakness related to c. diff and sepsis). No fall mentioned in the note.
“He was pulling a frozen chicken package out of freezer when it fell and struck his left lower leg anterior aspect”	Something else fell
“Had a fall on [DATE] with left knee laceration with minimal blood loss and no other injuries”	Note mentions prior fall (~ 3 weeks before) in HPI section, only as context for reason for visit (low hemoglobin counts)
“She has had multiple UTIs in the fall 2016”	Refers to the season “Fall”. Algorithm excludes for this, but only when written as “Fall of 20XX”
“PMH: Past Medical History...- s/p fall - ”	Algorithm mistakenly pulled this from past history section
“Patient has a known left-sided ovarian mass which her primary care physician and her husband falling.”	Transcription error in note (physician likely meant to write “following”)
“Pt states she fell apart after her divorce, that there is nothing that makes her happy”	Use of an idiom that contained the word fall
“The patient is unable to confirm falling”... “Son unaware and unable to confirm if patient fell or not”	Uncertainty about whether fall occurred; phrase “unable to confirm” was not included in algorithm for exclusion
“Negative for falls”	Negation term not included in algorithm
“Negative for back pain, falls and neck pain”	Negation term not included in algorithm
**False Negative**	
“...presents to the ED with complaints of left wrist pain sustained after a FOOSH injury earlier today while playing tennis.”	Use of acronym that describes the incident (‘Fall On OutStretched Hand’), rather than the word ‘fall’)
“Patient reports earlier this morning she was up in her bathroom attempting to use the toilet, sat down, missed the toilet and landed on her right hip.”	Word(s) other than “fall” used to describe fall incident in the note
“She was noted to have a syncopal event. She went down to the ground and struck her head...”; “...she syncopized at her aerobics class today. She does not remember the events immediately before or after.,, She did hit her head.”	Word(s) other than “fall” used to describe fall incident in the note
“He says that he intermittently woke up on the floor feeling unusual… it took him several minutes to realize that he had hit his head and that he had a large hematoma on the back left side of his head”	Word(s) other than “fall” used to describe fall incident in the note
“...presents with CT angiogram showing ‘occlusion’. Patient fell 1 month ago, had wound to her R knee. This opened into a sore, she has been managing this with her physician.”	Fall was not recent, but contributed to the cause of the ED visit.

^a^Ellipses added in place of deleted text

The most common reasons for false positive cases were the use of previously unseen fall-related words or phrases, not negated or excluded by the final test algorithm. These included “negative for”, “unable to confirm falling”, and “fell apart”. Other false positives resulted from fall terms being used to represent things other than the patient falling (e.g., a frozen chicken falling on the patient) or in a format not recognized by the algorithm as an exclusion (e.g. “fall 2016”, rather than the more-often used “fall of 2016”, already excluded in the algorithm). Two false positives were also the result of errors in the note/chart, one containing a transcription error incorrectly included the word “falling”, and one incorrectly including the word “fall” in the chief complaint, when the reason for the visit was something entirely different.

## Discussion

In the test dataset, the algorithm achieved recall and specificity in excess of 97% when compared to the gold standard consensus coded data. This performance was similar to that of the individual human abstractors when compared to the consensus code. The performance of coding-based definitions are difficult to estimate as these are often reported without validation [9–13] however likely significantly underestimate falls based on our earlier work involving chief complaint data [22].

NLP has been applied in the Emergency Department setting primarily in the setting of radiology reports for the identification of specific pathologies such as long bone fractures [23]. Rules-based NLP has been specifically used within the ED determine the presence on pneumonia in chest x-ray reports [24]. We are aware of one other NLP algorithm specifically aimed at detecting falls, however this had a different aim of finding all mentions of fall among many note types, as opposed to fall related visits among specific provider notes, and had a significantly higher false positive rate reported at ~ 7% [18].

Notably, our results were achieved with a simple, pragmatic rules-based approach. The potential for NLP to improve EHR phenotyping is well documented [15, 25], however significant barriers are perceived to implementing NLP derived algorithms to improve care, including need for specialized programming knowledge and large corpuses of annotated notes with which to train algorithms [16]. While statistical NLP approaches are in many ways more adaptable than rules-based approaches [26], our results highlight the ability of even simple programming solutions to interpret text for very specific tasks, achieving excellent performance without the need for a large training dataset. Our algorithm also has the advantage of transparency; given the simple rules-based format the function and anticipated output of the algorithm for a given input can be simply communicated to end users. These results suggest that a similar approach may be feasible for other ED presentations which are problematic to identify using discrete EHR data, such as concussion [27] and sepsis [28].

Given limitations in current methodology for identifying fall visits, implementation of this algorithm offers significant opportunity for increased ability to detect ED visits associated with falls [7, 22]. Potential applications for this include improvements in research methodology, quality measure development, and clinical patient identification. From a research standpoint, an easy-to- apply natural language processing definition can facilitate the conduction of high quality EHR based studies to examine pressing questions for geriatric emergency research, namely the characterization of current fall care and identification of patients at high risk of falling [7, 22].

Furthermore, reliable identification of fall phenotype without the need for manual abstraction offers the potential to create a denominator for quality measures to improve patient care. As older adults make up an increasing proportion of ED visits, national efforts are being made to improve and standardize geriatric care for older patients [29]. Quality measures are a key policy lever for enacting such improvement, and specific measures are lacking in the geriatric population, as well as for traumatic injuries [30]. Within the emergency department, quality measure development has been hampered by lack of ability to reliably identify patient cohorts based on presenting syndromes such as falls, as opposed to diagnosis codes, which are often applied after ED visits and subsequent care and may not reflect patient groups of interest to improving ED care [30, 31].

This identification strategy can additionally aid in epidemiologic surveillance applications: the ED is an important setting for measuring rates of injurious falls in communities, but currently these can only be captured by either coding-based definitions (which likely miss falls) [22] or via more time consuming survey or other manual abstraction based techniques [32]. Beyond epidemiology, the speed and computational simplicity of this algorithm would allow for potential insertion into EHR systems in real time to target patients for specific clinical interventions. Similar to current initiatives which are able to interpret text of radiology reports in real time to improve patient care [33], the ability to detect falls in real, or near real, time offers the potential to inform CDS tools to identify patients in need of further screening or potential referral for secondary prevention of future falls. As CDS use in the ED increases [34], incorporating real-time examination of text data has the potential to improve the precision and impact of these tools.

### Limitations

This work was completed using data from only a single center. While the concept could be adapted to other centers, the specific algorithm would need to be adapted to process notes formatted differently from those used within our system. Our algorithm did rely on excluding portions of the note which contained only historical information which would be difficult to distinguish from the present ED visit; this strategy was based on headers present in our notes and may need to be modified to adapt the algorithm to another site.

We used an iterative design process and ceased attempts to improve our algorithm when performance no longer increased in a meaningful sense from the training to testing dataset. Several misclassifications in our test dataset would be potentially preventable with further iterative updates to the algorithm; most notably the phrase “negative for falls” was not excluded from our process as this hadn’t occurred in the reviewed sections of the notes within our training dataset. Rarely used acronyms which indicate fall such as “FOOSH” for “fall on outstretched hand” could be added to the algorithm. In general, however, performing more testing iterations creates more rules and specific exceptions, adding to the complexity of the resultant program. The potential for rules and exceptions to interact in unpredictable fashion may limit the maximum effectiveness of a rules-based approach [26]. Other sources of misclassification, including typographical and transcription errors, would likely be very difficult to fix within the context of our rules-based approach.

## Conclusions

In this study, we demonstrated that a pragmatic algorithm was able to use provider notes to identify fall-related ED visits with excellent precision and recall. This finding offers promise not just for improving research methodology, but as a potential for identifying patients for targeted interventions, epidemiologic surveillance, and quality measure development.

## Additional file

Additional file 1: Conceptual Steps of Algorithm with Python Expressions. (DOCX 23 kb)

## Data Availability

The original data used in this study is comprised of notes which contain identifiable patient information and are not easily deidentified. Based on our IRB agreement, we are not able to share the original patient notes. The de-identified coded dataset of human and computer results by case is available from the corresponding author on reasonable request.

## Abbreviations

ED: Emergency Department
EHR: Electronic Health Record
NLP: Natural Language Processing
WHO: World Health Organization
