# Supplementary material for: Development and validation of a pragmatic natural language processing approach to identifying falls in older adults in the emergency department
Source: BMC Med Inform Decis Mak. 2019 Jul 22;19:138. doi: 10.1186/s12911-019-0843-7 (PMC6647058; doi:10.1186/s12911-019-0843-7)
Supplement: Supplementary file 1 — Conceptual Steps of Algorithm with Python Expressions. (DOCX 23 kb) [file 12911_2019_843_MOESM1_ESM.docx]

**Additional file 1: Conceptual Steps of Algorithm with Python Expressions**

1. Section note into sentences
2. Remove low-yield sections of notes
3. Look for fall expression within sentences in remaining notesections

Fall is indicated by a regular expression: ['f(a|e)ll(s|en|ing|ings)?']

1. Evaluate for “sure positives” phrases that indicate a fall definitely occurred

expression for “sure positives”: ["(before|after|prior to|while) (the )?fall(ing)?"]

(This code was added as these gerund phrase constructions were often preceeded by negation terms but did not indicate patient hadn’t fallen, for instance “patient didn’t hit head while falling”)

1. Evaluate for and remove exclusions:
2. Fallopian
3. Tube/line/catheter falling
4. Falling rate/level, or level/rate falling
5. Fall sustained/last month/year/names of months
6. Falling from/off/off of motorcycle/bicycle
7. Falling asleep
8. The phrase “frequent falls”
9. Not sure that/whether/if fall
10. “fall and return precautions”
11. Almost/nearly/would have fallen
12. Faint and fall (we have a clinic named faint and fall)
13. “Last fall” or “fall of”
14. Pression/systolic/MAP falling or falling Pressions/map/systolic
15. Sentences with construction “anticoagulant…… due to/because of …..fall risk”
16. Will/would/risk of ... fall
17. Falling off the wagon

Python constructions for above exclusions:

[

'fallopian', '(tube|line|catheter) f(a|e)ll(s|en|ing|ings)? out',

'(level|rate) f(a|e)ll(s|en|ing|ings)?',

'f(a|e)ll(s|en|ing|ings)? (\S+ )?(rate|level)',

‘(level|history of|by a|possible|may have) (\S+ )?f(a|e)ll(s|en|ing|ings)?',

'f(a|e)ll(s|en|ing|ings)? (sustained |last )?(\S+)?(month|year|jan(uary)? |feb(uary)?|mar(ch)?|apr(il)?|jun(e)?|jul(y)?|aug(ust)?|sep(tember)?|oct(ober)?|nov(ember)?|dec(em

ber)?)(s)?( ago)?', ‘f(a|e)ll(s|en|ing|ings)? (from|off|off of) (\S+ )?(motor|bi)cycle', 'f(a|e)ll(s|en|ing|ings)? asleep', 'f(a|e)ll(s|en|ing|ings)? clinic', 'frequent f(a|e)ll(s|en|ing|ings)?', 'f(a|e)ll(s|ing|en|ings)? level', 'not sure (that|whether|if) (\S+ )?f(a|e)ll(s|ing|en|ings)?', 'fall (and

)?(return )?precaution(s)?', "(almost|nearly|would have) f(a|e)ll(s|ing|en)?", 'faint (and|&) fall', 'last fall', 'fall of \d{4}', "(pressure|map |systolic) f(a|e)ll(s|ing|en)?", "f(a|e)ll(s|ing|en)?

(pressure|map(s)? |systolic)", "(AC|anticoagulation|lovenox|NOAC|warfarin|coumadin|rivaroxaban|heparin|eliquis|dabigatran|plav

ix|aggrenox|aspirin) (due to|because of)( increased| frequent | risk of)? f(a|e)ll(s|en|ing|ings)?(

risk)?",

"(will|would|risk of) f(a|e)ll(s|en|ing|ings)?",

"f(a|e)ll(s|en|ing|ings)? off the wagon”

]

1. Apply negation algorithm to all fall instances which are not “sure positives” which have not been excluded.

Negation algorithm: If a negation indicator appears before fall instance, the instance is negated; if the negation indicator appears after the instance it is ignored.

negation indicator expressions: ['no ', 'not ',"n't",'negative', 'never', 'denies', 'deny']

1. Aggregate all fall instances within a given note:

If positive instances > negative instances, mark note as positive for fall

If negative instances ≥ positive instances, mark note as negative for fall
